# Assessing the causal relationship between income inequality and mortality and self-rated health: protocol for systematic review and meta-analysis

**DOI:** 10.1186/s13643-022-01892-w

**Published:** 2022-02-03

**Authors:** Michal Shimonovich, Anna Pearce, Hilary Thomson, Gerry McCartney, Srinivasa Vittal Katikireddi

**Affiliations:** 1 MRC/CSO Social and Public Health Sciences Unit, University of Glasgow, Glasgow, United Kingdom; 2 College of Social Sciences, University of Glasgow, Glasgow, United Kingdom

**Keywords:** Income inequality, Causality, Bradford Hill, Self-rated health, Mortality

## Abstract

**Background:**

Income inequality has been linked to health and mortality. While there has been extensive research exploring the relationship, the evidence for whether the relationship is causal remains disputed. We describe the methods for a systematic review that will transparently assess whether a causal relationship exists between income inequality and mortality and self-rated health.

**Methods:**

We will identify relevant studies using search terms relating to income inequality, mortality, and self-rated health (SRH). Four databases will be searched: MEDLINE, ISI Web of Science, EMBASE, and the National Bureau of Economic Research. The inclusion criteria have been developed to identify the study designs best suited to assess causality: multilevel studies that have conditioned upon individual income (or a comparable measure, such as socioeconomic position) and natural experiment studies. Risk of bias assessment of included studies will be conducted using ROBINS-I. Where possible, we will convert all measures of income inequality into Gini coefficients and standardize the effect estimate of income inequality on mortality/SRH. We will conduct random-effects meta-analysis to estimate pooled effect estimates when possible. We will assess causality using modified Bradford Hill viewpoints and assess certainty using GRADE.

**Discussion:**

This systematic review protocol lays out the complexity of the relationship between income inequality and individual health, as well as our approach for assessing causality. Understanding whether income inequality impacts the health of individuals within a population has major policy implications. By setting out our methods and approach as transparently as we can, we hope this systematic review can provide clarity to an important topic for public policy and public health, as well as acting as an exemplar for other “causal reviews”.

## Introduction

Income inequality refers to the uneven distribution of income between people, assessed across regions, states, or countries [1, 2]. It is generally agreed that income inequality is associated with health outcomes at an ecological level. Our focus is on the more disputed hypothesis that after accounting for the impact of individual income, there is a relationship between income inequality and health [3]. This relationship is of great interest to public health and policymakers [3–5], though research evaluating the relationship has produced mixed results [6]. A recent systematic review of reviews evaluating the relationship between income inequality and health found considerable variation in the findings of thirteen systematic reviews, none of which were considered high-quality [2]. The extensive statistical heterogeneity observed by these reviews indicates that a causal relationship between income inequality and individual health remains unconfirmed. Understanding reasons for different effect estimates observed across studies (i.e. statistical heterogeneity or transportability as it is known in causal inference) is an important part of causal assessment [7, 8].

Systematic reviews, because of the scientific approach to selecting, appraising, and synthesising evidence, are useful for transparently bringing together a relevant body of evidence, evaluating statistical heterogeneity, and critically assessing risks of bias [9, 10]. A rigorous, systematic, and transparent evaluation of the evidence and causality is necessary to be certain of the relationship between income inequality and health, which can then clarify the need for policymakers to intervene on the exposure [11, 12]. The meta-analysis by Kondo and colleagues [13], though it was considered the highest quality identified in the review of reviews and incorporated multilevel data, did not include a critical appraisal of the literature nor assess the certainty of their findings (e.g. via a GRADE assessment). Pickett and Wilkinson [14] (also included in the review of reviews [2]) incorporated Bradford Hill viewpoints to their review assessing the relationship between income inequality and mortality. However, they also did not use a systematic approach to searching, identifying, analysing, evaluating, and synthesising evidence. Thus, we have not identified reviews that have incorporated a rigorous and robust systematic review process that incorporates causal assessment.

### Income inequality and health

**Fig. 1:**
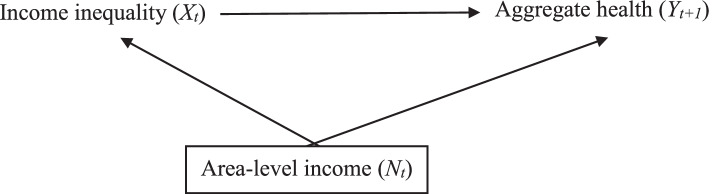
Directed acyclic graph (DAG) illustrating the relationship between income inequality (X), aggregate health (*Y*), and area-level income (*N*). Subscript *t* indicates the time

Early understanding of the relationship between income inequality and health was largely based on ecological studies [15–17]. The unit of analysis in these early studies was populations (usually countries or states), and though it is not described in this way, these studies also appear to argue that the relationship between income inequality and aggregate health is confounded by country/state-level income (e.g. GDP, GDP per capita). The directed acyclic graph (DAG)^1^ in Fig. 1 illustrates this relationship. There is a theorized non-linear relationship between previous area-level income and current aggregate health such that increasing area-level income increases aggregate health until a threshold where area-level income has no effect on aggregate health [20].

#### Area-level income confounding

Many ecological studies (and reviews of income inequality and health) address area-level income confounding (including related concepts such as GDP) by limiting the analysis to high-income countries (HIC) [16, 17] though it may not be appropriate to do so. These early ecological studies argued that the effect of area-level income confounding is hypothesized to be greater in low- and middle-income countries (LMIC) than in HIC [3]. However, recent evidence suggests the effect may be comparable in both HIC and LMIC [21]. In addition, considering the effect of income inequality on individual health in only HIC limits our understanding of the effect of income inequality on individual health in LMIC [22]. The extent to which area-level income is a confounding variable has also been debated. According to political economy theory, governmental policies affect income inequality which in turn affects future economic growth, regardless of the baseline level of inequality prior to government actions [23]. Economic growth affects current area-level income, such that area-level income may mediate the relationship between income inequality and aggregate health. However, the putative effect of income inequality on economic growth may depend on relative area-level income; the inverted-U hypothesis (known as the Kuznets curve [24]) suggests that the relationship between economic growth and income inequality is positive in LMIC and negative in HIC [25]. The empirical evidence regarding both the direction [25–27] and the sign of the relationship between income inequality and economic growth is mixed, with some arguing that the latter may be impacted by many other factors such as technological advancements [23, 25]. Thus, there is disagreement over the appropriateness of conditioning upon area-level income is an over-adjustment if it is a mediator rather than a confounder [22, 27].

#### Individual income confounding

**Fig. 2:**
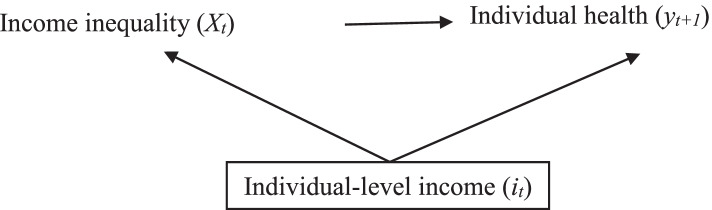
Directed acyclic graph (DAG) illustrating cross-level confounding of an individual’s income on the relationship between income inequality and individual health, annotated with subscript *t* to account for time. Area-level variables (e.g. income inequality, *X*) are capitalized while individual-level variables are lower-cased (e.g. individual-level income *i*) [31]. Individual income should be conditioned upon (represented by the square around individual-level income) to remove the confounding effect of individual income on the relationship between income inequality and individual health

With all other things being equal, a country with high-income inequality will likely have more low-income individuals (including a greater number of people in poverty i.e. falling substantially below the median income [28]) than a country with low-income inequality. The country with more low-income individuals will have disproportionally worse population health outcomes than the country with fewer low-income individuals. That is, the relationship between income inequality and individual health could entirely be explained by individual incomes [29]. Individual health, which has a non-linear relationship with individual income where the benefit of income for individual health depreciates with increasing income (known as the absolute income hypothesis) [30]. In other words, a £100 increase of income for low-income individuals (e.g. from £1000 to £1100) will produce a greater increase in health than a £100 increase for high-income individuals (e.g. from £100,000 to £100,100) [29]. Thus, individual income is a cross-level confounding variable [31] since it affects both the ecological exposure (i.e. income inequality is derived from individual incomes) and the individual-level outcome (individual-level health) [32] (see Fig. 2). Multilevel data, which include both aggregate- and individual-level variables, are necessary to disentangle the impact of individual income on income inequality and health and to evaluate the potential causal link [33].

### What is the mechanism by which income inequality causes individual health?

The psychosocial and neo-material frameworks offer two different, and often polarizing, explanations for the mechanisms through which income inequality affects individual health [5, 34]. While these frameworks are positioned as competing explanations [34], it is unlikely that either framework perfectly describes the mechanism between income inequality and individual health. While our systematic review does not aim to examine mechanisms, these can offer insight into the debate over which variables should be conditioned upon in any analysis. We use DAGs to articulate the different explanatory mechanisms described by each of these frameworks as they relate to our unit of analysis, individual health. Importantly, the DAGs are based both on our interpretation of key texts relating to each framework as well as our current understanding of the relationship structures. These frameworks were likely not written with causal thinking, as we know it today, in mind and thus illustrating these mechanisms through DAGs may not reflect the frameworks as they were originally intended.

#### Psychosocial framework

**Fig. 3:**
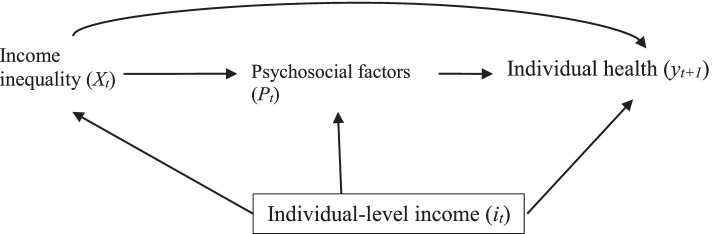
Direct acyclic graph (DAG) illustrating the relationship between income inequality and individual health mediated by psychosocial factors and confounded by individual income. Subscript *t* indicates time while area-level variables are capitalized and individual-level variables are lower-cased. This DAG reflects our general understanding of the psychosocial literature and is not intended to reflect the framework as described by any individuals [35]. According to the literature, psychosocial factors are theorized to mediate the effect of income inequality. Individual income is theorized to be a proxy for socioeconomic position, and some argue that conditioning upon individual income may be an over- adjustment that will underestimate the effect of income inequality on health. However, particularly as our outcome under study is individual health, we argue that individual income will not completely account for the individual social position (hence the line from individual income to psychosocial factors) and should be conditioned upon

According to the psychosocial framework (popularized by Richard Wilkinson and Kate Pickett in their book, *The Spirit Level* [35]), social status and hierarchies amongst individuals are more noticeable in countries with high-income inequality than in those with less income inequality [36]. Income inequality leads to social differentiation and comparisons, which has adverse psychological consequences (e.g. reduced social cohesion and trust; stress from comparing yourself to people who have more incomes and material goods; frustration and despair that could lead to crime and violence) which in turn negatively affects health [37–39]. The heightened awareness of hierarchy in high-income inequality societies is also hypothesized to impact individual behaviour, such that attenuated social cohesion increases the likelihood that individuals will smoke, consume alcohol and have diets that increase their risk of mortality [36]. The relationship between income inequality, psychosocial factors (such as social cohesion and behaviour), mortality, and individual income is summarized in Fig. 3.

As noted above, this DAG was developed to reflect how the psychosocial framework may be applied to our unit of analysis and our understanding of the impact of individual income on the relationship, and as a result, slightly diverges from some key elements of the original framework. Firstly, the outcome under study here is individual health, not aggregate health as originally described [38, 40]. Wilkinson, both in his early work on income inequality and life expectancy [17] and in his later work with Pickett when the psychosocial framework was formalized, has been criticized [41–43] for relying on ecological studies to make assumptions about individual health. They both disputed this claim and insisted that findings from ecological studies were only used to understand the relationship between income inequality and population health [14, 44].

The second key element of the psychosocial framework altered in our DAG is conditioning upon individual income. The psychological framework posits that individual income should not be conditioned upon, even if the unit of analysis is individual health, because it is a proxy for psychosocial consequences of social differentiation and comparison (which mediate rather than confound the relationship between income inequality and health) [44]. However, we argue that individual incomes (and other socio-economic characteristics such as socioeconomic position (SEP)) are poor proxies for psychosocial factors and should be conditioned upon as candidate confounders (as has been done in several studies [45–47] and reviews [13, 48]).

#### Neo-material framework

**Fig. 4:**
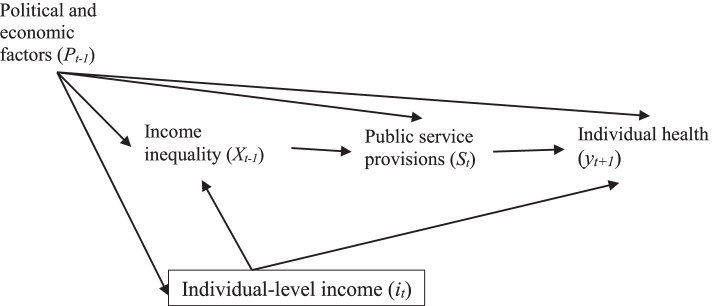
Directed acyclic graph (DAG) of the simplified relationship between income inequality and individual health, confounded by individual income, political and economic factors and mediated by public service provisions. Time is indicated with a subscript *t* while area-level variables are capitalized and individual-level variables are lower-cased. This DAG reflects our general understanding of the neo-material literature, though the structure of the relationship (including whether public service provisions are a co-exposure and thus should be conditioned upon, not shown) remains debated

The neo-material framework hypothesizes that political and economic actions affect income inequality and public resources, such that individuals with fewer resources are disproportionately disadvantaged in countries with high-income inequality [5]. Political and economic actions can include taxes, cash transfers, political structure, and power of organized labour. In our interpretation of early work on the neo-material framework [5] (see Fig. 4), political and economic factors are an upstream confounder of the relationship between income inequality and individual health and while individual income is also a confounder, it is downstream of political and economic factors. In addition to income inequality, political and economic factors affect both public service provisions (on the pathways between income inequality and health) and individual income (e.g. cash transfers). The neo-material framework highlights why time lags may be necessary to understand the effect of income inequality on individual health [3]. For example, it may take as much as fifteen years [49] for the effect of limiting organized labour’s power to increase income inequality and adversely impact health.

We also argue that political and economic actions can modify the effect of income inequality on individual health through public service provisions (e.g. strength labour unions to improve access to healthcare). However, it is important to note that the interpretation in Fig. 4 may not accurately represent the relationship; it may be that public service provisions are co-exposures of individual health and occur alongside income inequality [50] (i.e. without the arrow to income inequality shown in the DAG).

## Methods

Information in this systematic review protocol is reported according to the Preferred Reporting Items for Systematic Review and Meta-Analysis Protocols (PRISMA-P) guidelines [51]. The systematic review was submitted to PROSPERO (CRD42021252791) and any subsequent amendments to the protocol will be documented and published in the systematic review.

### Aims and objectives

The main aims of the systematic review are to understand if there is a relationship between income inequality and individual mortality and/or SRH, and, if so, if that relationship is causal. While our aims do not include understanding the putative causal mechanism, we will consider evidence of a causal mechanism to strengthen our certainty that a causal relationship exists. To address these aims, we will attempt to address the following questions:

1. What evidence is there of a relationship between income inequality and individual mortality and/or SRH?
2. What is the magnitude of the association between income inequality and individual mortality and/or SRH?
3. To what extent does the available evidence support a causal relationship between income inequality and individual mortality/SRH?

### Study design

To answer our research questions, this systematic review will incorporate approaches to causal assessment. We will apply adapted Bradford Hill viewpoints where two viewpoints (coherence and analogy) have been excluded. Our interpretation and approach to applying the viewpoints, including which viewpoints to use, is based on earlier research comparing different approaches to causal assessment [52] and a scoping review of causal systematic reviews (unpublished). These interpretations were used to inform our search strategy and inclusion and exclusion criteria. The viewpoints, their interpretation, and the evidence we will use to determine if a viewpoint is met are in Table 1.

**Table 1:** Causal assessment approach

Viewpoint	Interpretation	Type of evidence to assess each viewpoint	Evidence considered to determine if viewpoint has been “met”
Strength of association	Our scoping review of causal assessment found a range of effect sizes that were considered strong (e.g. RR > 1.20 and RR > 5.0). For GRADE, a strong association is an RR between 2–5 while a very strong is an RR greater than 5 [53]. We will extract the effect sizes from studies that conditioned upon individual income and, when synthesized, will note if it is considered “strong” according to GRADE. However, we will consider those alongside any information on residual confounding (i.e. the likelihood that confounding effects remain after conditioning), unmeasured confounding or evidence of over-adjusting such that the effect size is underestimated (see Table 4 for list of confounding variables).	Cohort and cross-sectional studies with multilevel modelling	1. Rather than focus on whether an effect size falls above a specific size, we will prioritize evidence that residual confounding and/or unmeasured confounding accounted for (including measure and assessment of the *E*-value, i.e. the minimum observed association that would unlikely be explained by a confounding variable [54]).
Consistency	Our scoping review found that reviews applying Bradford Hill viewpoints and considering consistency often aimed to understand if effect estimates were consistent across populations, settings or study designs. However, we will apply the principles of a realist review that focus on explaining why effect sizes may be similar or differ rather than determining if they are consistent. We will account for transportability (i.e. to what extent can causal effect in one context be applied to another) and what factors that undermine transportability help explain statistical heterogeneity across studies. If necessary, we will use DAGs to illustrate our assumptions about what factors (such as studies from the US) undermine transportability and how.	Cohort and cross-sectional studies with multilevel modelling Natural experiments	1. Explanations for differences in effect sizes (articulated in a DAG) (see Table 4 for possible explanations). 2. Evidence that effect estimates are consistent across different settings and populations (especially if there is evidence that bias in these studies have been addressed).
Temporality	Evaluating a relationship’s temporality (i.e. if the exposure under study came before the outcome under study) involves assessing the evidence for reverse causation. Thus, longitudinal data are required to understand if a relationship between income inequality and health is observed even after conditioning upon individual health *prior* to changes in income inequality.	Cohort studies with multilevel modelling Natural experiments	1. Health outcomes happened after income inequality change.
Specificity	We do not anticipate a lot of evidence to support a specific (i.e. one-to-one) relationship between income inequality and individual health. However, if we identify studies that look at falsification outcomes or exposures (i.e. variables associated with the confounding variable but not with the exposure or outcome under study, respectively), these will strengthen our certainty of a causal relationship. We will use DAGs to articulate our assumptions of falsification outcomes or exposures.	Cohort and cross-sectional studies with multilevel modelling	1. Evidence confounding variables were adequately conditioned upon using falsification outcome/exposures.
Dose-response	Evidence of a dose-response relationship may not be as useful in causal assessment as is commonly assumed [52], particularly if the impact of confounding variables is not considered alongside a dose-response gradient (as is the case with the strength of association). Because of this, we will only evaluate dose-response if we can identify studies that have taken confounding by individual-level income into account.	Cohort and cross-sectional studies with multilevel modelling	1. Evidence of a dose-response relationship within studies that have accounted for individual-level income.
Plausibility	Our scoping review found that many reviews considered a relationship plausible if a credible mechanism could be identified (though what constitutes as “credible” was not clarified). There are two well-known mechanisms explaining the relationship between income inequality and individual health: (1) psychosocial factors and (2) neo-material factors. While it is beyond the scope of the SR to determine which of these mechanisms is most plausible, we will note any empirical evidence that does examine mechanisms and narratively synthesize their findings.	Cohort and cross-sectional studies with multilevel modelling Natural experiments	1. Empirical evidence (if any) that explains the mechanism by which income inequality causes individual health, to be synthesized narratively.
Experiment	Experimental evidence is considered amongst the most important for causal inference. We will consider natural experiments (multilevel and ecological cohort studies) to assess experimental support of causality. Two reviews from our scoping review used the MRC guidance on natural experiments to compare findings from observational data using different analytical methods and study designs to account for bias and emulate randomized studies. We will similarly compare the findings of studies using different methods.	Natural experiment	1. Evidence of an effect from natural experiment studies which better account for confounding than traditional observational studies. 2. Consistent findings from natural experiment studies using different methodological approaches.

### Eligibility criteria

The inclusion and exclusion criteria for our review are summarized in Table 2.

**Table 2:** Inclusion and exclusion criteria

Category	Inclusion criteria	Exclusion criteria
Study design	All cohort studies using multilevel data that consider income inequality and all-cause mortality or SRH We will also include all natural experiment studies that consider income inequality and all-cause mortality. Multilevel studies will be included if they report at least two levels and have conditioned upon individual income or another measure of individual socioeconomic position.	1. Individual-level studies (i.e. those that evaluate the relationship between individual income and individual mortality). 2. Studies that do not condition upon individual-level measure of income or socioeconomic position.
Population	We will include studies with an adult population. We will not limit studies based on relative income (e.g. restrict studies to only high-income countries) but will note differences in effect estimates for studies that do condition upon area-level income (see Table 4)	3. Majority (i.e. >/50%) of the study population is under eighteen years old.
Intervention/exposure	All measures of income inequality, including the Gini coefficient, the ratio of incomes between high-income individuals/earners to low-income individuals/earners, the Theil index or the share of income that is earned by high-income individuals [55]. We will not exclude based on measures of income inequality but will discuss the potential impact of different income inequality measures on statistical heterogeneity. We will also not exclude based on the quality of studies but will note differences according to our risk of bias assessment (see Table 4).	4. Studies that do not measure income inequality.
Comparator	All comparator types will be included.	None.
Outcome	All-cause mortality (including mortality rates and life expectancy) and SRH will be included as common indicators of health [56]. SRH is typically a response to a single-item question about general health which has been shown to be a reliable predictor of mortality [57]. Response categories are usually based on a Likert scale, with four or five categories most commonly used and the variable analysed as either binary or continuous [58].	5. Specific causes of mortality or specific health outcomes, such as mental or physical health only OR wellbeing or happiness (rather than SRH).
Setting	All area-types (e.g. municipalities, states, provinces, regions, or countries) will be included and differences due to type of area-level will be accounted for in the synthesis.	None
Year	We will search for and include all studies from 1992, based on the first Wilkinson study to suggest a relationship between income inequality and mortality based on ecological data [17], to present. However, we do not expect to identify multi-level studies published before 1996, as noted in the most recent meta-analysis of multi-level studies in income inequality and mortality and SRH [13].	6. Studies prior to 1992.
Follow-up	We will not limit studies based on follow-up time between income inequality intervention/exposure and outcome.	None.

### Search, screening, data extraction, and critical appraisal

#### Search strategy

We developed our search strategy with the support of an information scientist. Our strategy was based on two categories of keywords: income inequality (e.g. “income”, “inequality”, “income inequality”, “Gini”) and health (e.g. “mortality”, “all-cause”, “life-expectancy”, “death”, and “health”). We will search MEDLINE, ISI Web of Science, EMBASE, and the National Bureau of Economic Research. The search strategy is in Appendix. We do not plan to search grey literature but references in all included studies will be checked to retrieve any additional articles missed by our search strategy.

#### Study selection process

Studies identified from the search will be imported into Covidence where they will be assessed against the inclusion and exclusion criteria. One reviewer (MS) will screen all titles to identify and exclude references that obviously do not meet both the exposure and outcome criteria. An initial “title only” screening is a time-efficient approach that is unlikely to affect the amount of relevant included studies [59]. Subsequently, two reviewers will independently screen titles and abstracts of all remaining references. Studies will be included if the study design is unclear but all other criteria are met. Of those that potentially meet the inclusion criteria, full-texts will be identified and independently screened by two reviewers. The reasons for excluding full-text studies will be recorded and a third reviewer will resolve disagreements.

#### Data extraction

One reviewer will extract data from included studies. A second reviewer will check all of the extracted data. We will extract study information, the indicators used to measure income inequality and morality/SRH and baseline information in the population include sex, socioeconomic status, ethnicity, and age. We will note if the studies explain why certain variables were conditioned upon. A sample data extraction form is provided in Table 3.

**Table 3:** Data extraction information

Category	Information to be extracted
Study information	Author, year Country Name of study Sample size Age Sex (% female/male) Overall conclusion on income inequality and individual health
Exposure	Measure of income inequality used
Outcome	Measure of association (e.g. RR, HR, ORs) Measurement of mortality or self-reported health Number of cases
Confounding variables	Individual-level confounding variables (such as individual or household income) Data sources for individual-level confounding Area-level confounding variables and data sources (if area-level variables were conditioned upon)
Statistical analysis	Method of analysis
Additional factors impacting statistical heterogeneity/transportability	Area type (country, state, municipality, etc.) US or non-US Population density

#### Risk of bias assessment

Two reviewers will independently assess the included studies for bias using the ROBINS-I tool for observational data [60]. For ROBINS-I, we have developed a target trial that considers exposures, comparisons, confounding, and co-exposures. Confounding variables are those that cause both the exposure and outcome under study, while co-exposures are those that may be received alongside the exposure understudy and may be associated with (though not necessarily the cause of) the exposure and outcome under study [60]. This is helpful when considering if studies conditioned upon variables that we have considered mediators (and should not be conditioned upon) and those that we have considered confounding variables (and should be conditioned upon) [61]. Though ROBINS-I has been criticized for low interrater reliability [62] and is often misapplied [63], it facilitates thorough and systematic methodological evaluation of non-randomized studies using principles of causal thinking [64]. This methodological evaluation will be used to inform considerations of certainty through GRADE and causal assessment using the adapted Bradford Hill viewpoints.

### Strategy for synthesis and causal analysis

Our method for data analysis includes both a meta-analysis as well as causal analysis. As it is unlikely we will be able to test for political and economic factors, we will narratively describe the differences across the included studies.

#### Meta-analysis

We will conduct a meta-analysis to determine the overall association between income inequality and mortality as well as a separate meta-analysis of and income inequality and SRH. Where possible, we will synthesize the outcome measures for mortality (mortality rates, life-expectancy) and SRH (SRH, wellbeing). All meta-analyses will be random-effects, given the considerable heterogeneity expected across contexts, study designs, and populations. If possible, we will convert all of the indicators into Gini coefficients and standardize the effect estimate of income inequality on mortality/SRH. as this is likely to be the most commonly used indicator for income inequality [13]. In addition, we will explore factors we believe may account for statistical heterogeneity (see Table 4) as part of understanding the causal relationship.

**Table 4:** Target trial characteristics for ROBINS-I risk of bias

Exposure	Area (any size, type, population size) with income inequality
Comparator	Comparable area size, type, population size with low-income inequality
Outcome	Health outcomes (mortality, self-rated health)
Confounding variables	Individual income Socioeconomic position
Co-exposures	Tax system Strength of organized labour Universal healthcare
Mediators	Psychosocial factors
Factors that may undermine transportability/ explain statistical heterogeneity (based partly on [3, 13]).	• Gini vs non-Gini coefficient measure for income inequality • Time lag between exposure and outcome measurement • US vs non-US studies • Within country vs between country comparisons • Area type, size, and population size • Relative income inequality (e.g. Gini above vs below threshold) • Area level income • Education

#### GRADE

We will perform a GRADE assessment, a widely adopted approach for assessing the certainty of evidence across a body of evidence, on both outcomes (mortality and SRH) [65]. The GRADE assessment of certainty, which describes the confidence in the effect estimates [65] is based on the assessment of risk of bias [66], indirectness of included evidence [67], imprecision [68], inconsistency [69], and publication bias across the body of evidence [70]. Certainty from non-randomized studies (NRS), which are the most likely types of studies included in this review, are automatically rated at the same level (“high”) as randomized controlled trials when using ROBINS-I, and then subject to downgrading based on a set of domains [71]. Large associations, dose-response relationships, and adjusting for plausible confounding upgrade certainty of the evidence for NRSs.

#### Causal assessment

We will assess the studies to explore the causal relationship between the income inequality and mortality. Table 1 provides an overview of the evidence we will consider for each Bradford Hill viewpoint. Together with the meta-analysis, we will narratively describe our confidence, based on the evidence, that there is a relationship between income inequality and individual health and our confidence that it is causal.

## Conclusion

This protocol describes the methods for conducting a causal systematic review on income inequality and individual health. In the first section, we highlighted the debate over the nature of the relationship and explain consequences for observed associations. While testing the two explanatory, and often competing, mechanisms are beyond the scope of this systematic review, we are explicitly favouring the neo-material framework by both including multilevel studies that condition upon individual income and considering political and economic factors as co-exposures. We expect the complexities of standardising and synthesising evidence from a wide range of studies using different income inequality measurements and statistical methods to be amongst the most challenging aspect of this review. We hope this review will not only elucidate the relationship between income inequality and health but also act as an exemplar for transparently performing causal assessment in evidence synthesis.

## Data Availability

Data sharing is not applicable to this article as no datasets were generated or analysed during the current study.

## Appendix

Search strategy for MEDLINE and EMBASE (conducted January 2021)

1. Socioeconomic Factors/
2. Income/
3. (individual adj1 income).ab,ti.
4. (income inequality or income inequity).ab,ti.
5. (gini adj (index or ratio or coefficient)).ab,ti.
6. health disparities.ab,ti.
7. Mortality/
8. “Cause of Death”/
9. Life Expectancy/
10. life expectancy.ab,ti.
11. Health/
12. 1 or 2 or 3 or 4 or 5 or 6
13. 7 or 8 or 9 or 10 or 11
14. 12 and 13
15. limit 14 to yr=”1992 - 2020

## Footnotes

1 Our DAGs are purposefully simplified and are not likely to capture the complexity of the relationships under study. DAG do not illustrate relationship signs (e.g. positive vs negative), magnitudes (e.g. effect sizes), or shapes (e.g. linear, monotonic) [18]. Moreover, as DAGs do not illustrated cyclical relationships [19], variables are annotated with a subscript of t to indicate time points to distinguish the time when variables are observed.
